# Endoscopic resection for an esophageal inclusion cyst

**DOI:** 10.1055/a-2094-9279

**Published:** 2023-06-15

**Authors:** Lifan Zhang, Huanhuan Yang, Liansong Ye, Ou Chen, Bing Hu

**Affiliations:** 1 Department of Gastroenterology and Hepatology, West China Hospital, Sichuan University, Sichuan, China; 2 Department of Gastroenterology, Ya’an People’s Hospital, Sichuan, China

A 24-year-old woman complained of intermittent dysphagia for 2 years. She reported a previous medical history of asthma, hyperthyroidism, and myocarditis. Physical examination revealed no special abnormalities. Computed tomography detected a 5.2 × 4.2-cm mass with a clear boundary in the esophagus ( Fig. 1 a ). Endoscopy showed a large lesion 30–34 cm from the incisors ( Fig. 1 b ). Endoscopic ultrasonography confirmed the hypoechoic lesion with hyperechoic foci, originating from the submucosal layer ( Fig. 1 c ). Endoscopic resection was performed for this patient ( Fig. 2 , Video 1 ). After submucosal injection at 3 cm proximal to the lesion, a mucosal incision was made using a dual knife. A submucosal tunnel was subsequently created. When the lesion was partially exposed with an insulated-tip knife, it ruptured suddenly and yellow milky fluid flowed out, suggesting an esophageal cyst. The top layer of the cyst was resected using the dual knife. Then, the remaining rest cyst wall was destroyed using electrocoagulation forceps and anhydrous alcohol. The mucosa beyond the cyst was also removed using a snare. There was no bleeding or perforation during the procedure. Histopathology showed a pseudostratified ciliated columnar epithelium-lined cyst wall and no cartilage or bilayer smooth muscle, confirming it was esophageal inclusion cyst. The patient recovered uneventfully. During 2 months of follow-up, the patient reported no further discomfort; endoscopy also showed complete healing of the wound ( Fig. 3 ).

**Fig. 1:**
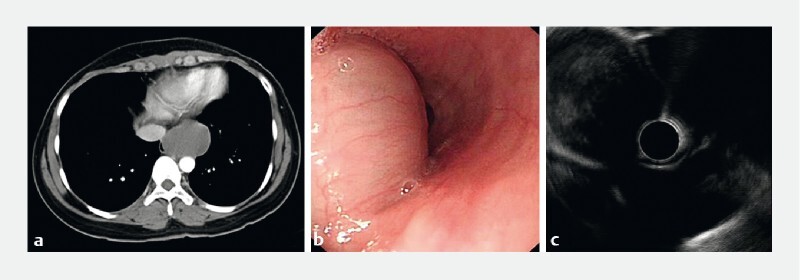
Preoperative images. **a** Computed tomography showed a soft-tissue mass in the esophagus. **b** Endoscopy showed a large lesion 30–34 cm from the incisors. **c** Endoscopic ultrasonography showed a hypoechoic lesion with hyperechoic foci originating from the submucosal layer.

**Fig. 2:**
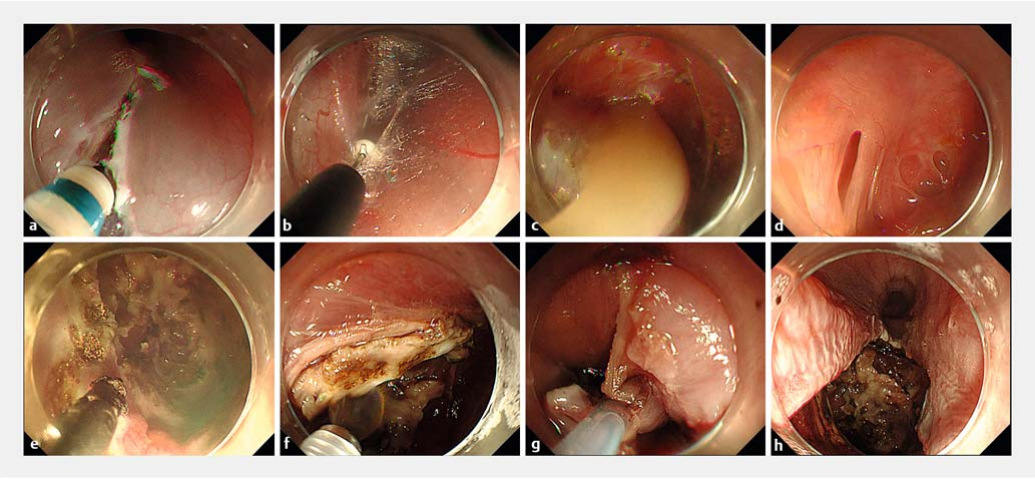
The process of endoscopic resection. **a** Mucosal incision using a dual knife after submucosal injection. **b** Lesion dissection using an insulated-tip knife. **c** Yellow milky fluid flowing out after lesion rupture. **d** The base of the lesion after lesion unroofing and fluid suction. **e** Electrocoagulation forceps applied for destruction of the wall. **f** Spraying anhydrous alcohol for destruction of the wall. **g** Removal of the mucosa beyond the lesion using a snare. **h** The wound without closure.

**Video 1** Endoscopic resection for an esophageal inclusion cyst.


**Fig. 3:**
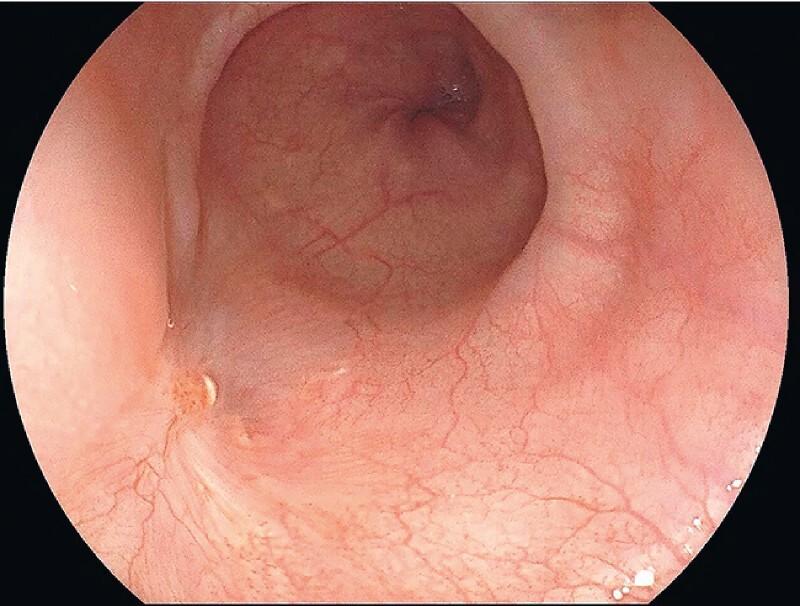
Endoscopic follow-up showed complete healing of the wound.

Esophageal cysts are rare, with an incidence of one in 8200 1 . Compared with duplication and bronchogenic cysts, an inclusion cyst is characterized by the absence of bilayer smooth muscle and cartilage 2 . All these esophageal cysts are usually benign and asymptomatic, but they may cause dysphagia. In addition, malignant transformation of esophageal cysts has also been reported 3 . Removal of esophageal cysts usually completely relieves symptoms or complications. The key point is to completely remove the entire cyst wall to prevent recurrence.

Endoscopy_UCTN_Code_TTT_1AO_2AG

## Notes

Endoscopy E-Videos

https://eref.thieme.de/e-videos
*E-Videos* is an open access online section of the journal *Endoscopy* , reporting on interesting cases and new techniques in gastroenterological endoscopy. All papers include a high-quality video and are published with a Creative Commons CC-BY license. Endoscopy E-Videos qualify for HINARI discounts and waivers and eligibility is automatically checked during the submission process. We grant 100% waivers to articles whose corresponding authors are based in Group A countries and 50% waivers to those who are based in Group B countries as classified by Research4Life (see: https://www.research4life.org/access/eligibility/ ). This section has its own submission website at https://mc.manuscriptcentral.com/e-videos
